# Luspatercept restores SDF-1-mediated hematopoietic support by MDS-derived mesenchymal stromal cells

**DOI:** 10.1038/s41375-021-01275-5

**Published:** 2021-05-17

**Authors:** Manja Wobus, Anna Mies, Nandini Asokan, Uta Oelschlägel, Kristin Möbus, Susann Winter, Michael Cross, Heike Weidner, Martina Rauner, Lorenz C. Hofbauer, Martin Bornhäuser, Uwe Platzbecker

**Affiliations:** 1 Department of Medicine I, University Hospital Carl Gustav Carus, Technische Universität, Dresden, Germany; 2 German Consortium for Cancer Research (DKTK, Site Dresden), DKFZ, Heidelberg, Germany; 3 Medical Clinic and Policlinic I, Hematology and Cellular Therapy, University Hospital Leipzig, Leipzig, Germany; 4 Department of Medicine III & Center for Healthy Aging University Hospital Carl Gustav Carus, Technische Universität, Dresden, Germany; 5 National Center for Tumor Diseases NCT/UCC, Dresden, Germany

**Keywords:** Haematopoietic stem cells, Stem-cell research, Translational research

## Abstract

The bone marrow microenvironment (BMME) plays a key role in the pathophysiology of myelodysplastic syndromes (MDS), clonal blood disorders affecting the differentiation, and maturation of hematopoietic stem and progenitor cells (HSPCs). In lower-risk MDS patients, ineffective late-stage erythropoiesis can be restored by luspatercept, an activin receptor type IIB ligand trap. Here, we investigated whether luspatercept can modulate the functional properties of mesenchymal stromal cells (MSCs) as key components of the BMME. Luspatercept treatment inhibited Smad2/3 phosphorylation in both healthy and MDS MSCs and reversed disease-associated alterations in SDF-1 secretion. Pre-treatment of MDS MSCs with luspatercept restored the subsequent clonogenic potential of co-cultured HSPCs and increased both their stromal-adherence and their expression of both CXCR4 and ß3 integrin. Luspatercept pre-treatment of MSCs also increased the subsequent homing of co-cultured HSPCs in zebrafish embryos. MSCs derived from patients who had received luspatercept treatment had an increased capacity to maintain the colony forming potential of normal but not MDS HSPCs. These data provide the first evidence that luspatercept impacts the BMME directly, leading to a selective restoration of the ineffective hematopoiesis that is a hallmark of MDS.

## Introduction

Myelodysplastic syndromes (MDS) are clonal hematopoietic stem/progenitor cell (HSPC) disorders characterized by ineffective hematopoiesis leading to anemia, leukopenia, and an increased risk of transformation into acute myeloid leukemia [1, 2]. Accumulating evidence suggests that the pathophysiology of MDS is strongly influenced by the bone marrow microenvironment (BMME) [3], in which mesenchymal stromal cells (MSC) actively support HSPCs via direct cell–cell contacts, the production of extracellular matrix or the exchange of soluble factors [4].

The ineffective erythropoiesis typical of MDS has been linked to Smad2/3 signaling in response to increased levels of transforming growth factor (TGF)-ß family ligands, including growth differentiation factors (GDFs), activins, bone morphogenic proteins (BMPs), and TGF-β [5, 6]. Smad-dependent BMP signaling affects the production of SDF-1 in the BMME, reducing HSPC homing, engraftment, and mobilization [7], while Smad2 inhibition stimulates hematopoiesis from primary MDS progenitors [6].

Luspatercept (ACE-536) is a novel, recombinant fusion protein comprising modified activin receptor type IIB linked to the Fc domain of human immunoglobulin G1. It binds to selected TGF-β superfamily ligands including GDF-11 and activin B, restores late-stage erythropoiesis in MDS, and has become a first-in-class erythroid maturation agent [8]. Recently, luspatercept has been shown to increase hemoglobin levels in LR-MDS patients in both a phase 2 (MDSNCT01749514; NCT02268383) and a phase 3 randomized, double-blind, placebo-controlled study (NCT02631070) [9, 10] in which 63% of patients showed erythroid responses with 38% achieving transfusion independence ≥8 weeks [11]. Similarly, the murine luspatercept analog RAP-536 promoted late-stage erythrocyte maturation and led to rapid, sustained, dose-dependent increases in hemoglobin and red blood cell concentrations in a murine model of MDS [8].

Although the clinical benefits of luspatercept on ineffective erythropoiesis are evident, its impact on the BMME has not been investigated in any detail. To approach this question in vitro, we assessed the effects of luspatercept and GDF-11 on Smad signaling and hematopoietic support capacity of MSCs from MDS patients and age-adjusted healthy controls.

To validate the results obtained for co-cultured HSPCs in vivo, we chose the zebrafish (Danio rerio) model, which offers important advantages for studies of stem cell migration. As an accessible vertebrate, the zebrafish has a tissue and organ complement similar to that of mammals and has proven to be an excellent model to study adult HSPCs in the transplant setting [12, 13].

We show that luspatercept treatment of MDS MSCs inhibited Smad2/3 phosphorylation and restored SDF-1 secretion. Subsequently, this increased the yield of stroma-associated normal HSPCs and their expression of the SDF-1 receptor CXCR4 and of β3 integrin. Moreover, clonogenic potential and migratory capacity both in vitro and in vivo were higher for HSPCs cultured on MSCs pre-treated with luspatercept than for those cultured on untreated controls. MSCs derived from luspatercept-treated patients had an increased capacity to maintain the colony forming potential of normal but not MDS HSPCs.

## Materials and methods

### Patients

Heparinized BM samples were obtained at standard diagnostic aspiration from untreated MDS patients mostly with low or intermediate risk disease (*N* = 19, age 27–79 years, patients’ characteristics are depicted in Supplementary Table 1) and from hematologically healthy donors undergoing hip replacement surgery (*N* = 10, age 45–77 years). Institutional Review Board approval and written informed consent was obtained for all procedures. MSCs were prepared from both sources and BM plasma was collected from selected MDS patients (*N* = 10).

### Cell culture

Primary MSCs from passages 2–5 were used for the experiments. Subconfluent cultures were treated with 0.1 µg/ml GDF-11 (Biomol, Hamburg, Germany) and 10 µg/ml RAP-536 (provided by Celgene/Acceleron) for up to 1 week. Cell growth and proliferation were calculated by cell counting with trypan blue.

HSPCs were isolated using CD34 antibody-conjugated magnetic beads, according to the manufacturer’s instructions (Miltenyi Biotec, Bergisch Gladbach, Germany). The purity of the isolated CD34+ population was confirmed by flow cytometry and only fractions >95% positivity were used in the analysis.

For co-cultures, MSCs were pre-treated with GDF-11 and/or RAP-536 in DMEM/10% FCS for 7 days. For co-culture, CD34+ cells were added in CellGro medium (CellGenix, Freiburg, Germany) containing SCF, FLT3-L and IL-3 (10 ng/ml each, all from Miltenyi Biotec). After 7 days of co-culture, supernatant cells were collected, the stromal layer with adherent HSPCs was trypsinized, and the number of CD45+, CD61+, and CD184+ cells was evaluated in both fractions by flow cytometry.

### Clonogenic assays

Cobblestone area forming-cell (CAF-C) assays were performed over 4 weeks using pre-treated healthy or MDS MSC layers. One thousand magnetically isolated CD34+ cells were added in StemMACS HSC-CFU complete medium (Miltenyi Biotec).

Colony forming unit (CFU) assays were carried out using cells harvested after 1 week of co-culture, with 300 cells being plated in enriched methylcellulose medium with recombinant cytokines (MethoCult H4435, Stem Cell Technologies). Colonies were counted after 2 weeks and classified under a microscope or with the StemVision system (Stem Cell Technologies).

### Statistical analysis

Statistical analysis was performed using GraphPad Prism software version 6.04 (GraphPad Software). Data are presented as mean ± standard deviation (SD). All experiments were repeated between three and five times. For multiple group comparisons, ANOVA followed by Tukey’s or the Sidak post-hoc test as well as Kruskal–Wallis followed by Dunn’s multiple comparisons post-hoc test was performed. A *p* value of less than 0.05 was regarded as being statistically significant.

### Additional methods

Additional methods are described under Supplementary information.

## Results

### Luspatercept/RAP-536 modulates Smad2/3 signaling and Smad4 expression in MSCs

**Fig. 1:**
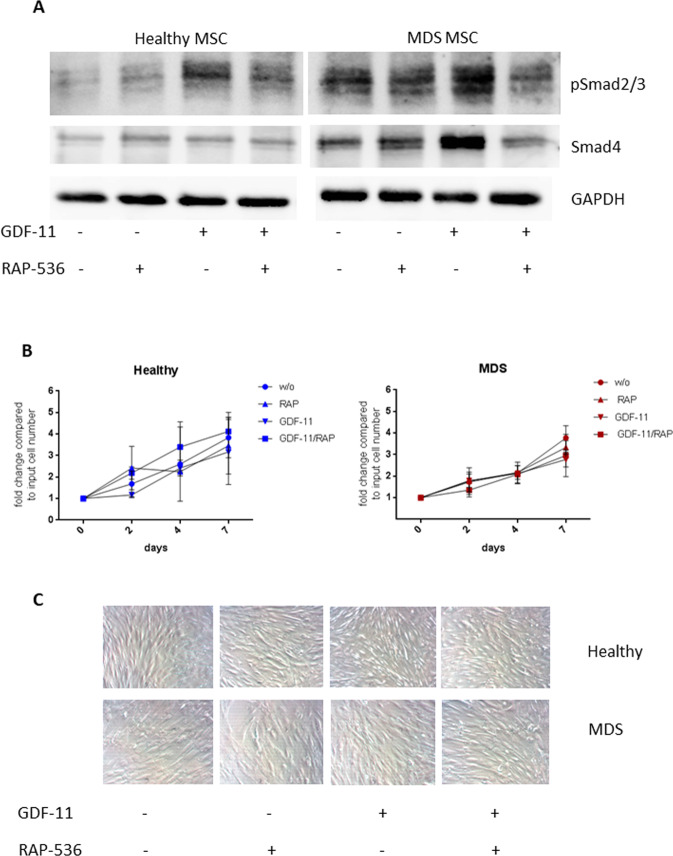
Smad2/3 and Smad4 activation by GDF-11 in human MSCs are inhibited by RAP-536 while neither treatment alters MSC growth or proliferation. **A** Western blot analysis of phospho Smad2/3 (52 kDa) and Smad4 (60 kDa) in healthy and MDS MSCs following 0.1 µg/ml GDF-11/ 10 µg/ml RAP-536 treatment. GAPDH (37 kDa) served as reference protein. **B** Cell counts of untreated (w/o) or GDF-11/RAP-536-treated healthy and MDS MSCs were determined by trypan blue staining after 2, 4, and 7 days. Values represent mean ± SD in triplicate for *N* = 3 healthy and *N* = 5 MDS MSC cell counts normalized to the starting cell number. **C** Representative light microscopy images showing the morphology of healthy (upper panel) and MDS (lower panel) MSCs at indicated treatment conditions at day 7 (magnification 20×).

Standardized conditions for the in vitro treatment of human MSCs with GDF-11 and the luspatercept homolog RAP-536 were established on the basis of Smad2/3 phosphorylation levels. Recombinant GDF-11 at a concentration of 0.1 µg/ml was found to induce Smad2/3 signaling in both healthy and MDS MSCs, while a concentration of 10 µg/ml RAP-536 was sufficient for 50% inhibition of Smad2/3 phosphorylation in GDF-11-treated cells. These concentrations were used in all subsequent experiments. Importantly, baseline phospho Smad2/3 levels were consistently higher in untreated MDS MSCs than in healthy controls (Fig. 1A). The levels of Smad4, which shuttles to the nucleus and may directly affect target gene expression by binding to cognate sites in target promoters, reflected those of phospho Smad2/3 with higher basal levels in MDS MSCs (Fig. 1A).

The proliferation rate was lower in MDS MSCs than in healthy controls, but was not significantly altered by GDF-11/RAP-536 treatment (Fig. 1B). MDS MSCs also displayed a more heterogeneous morphology (Fig. 1C), in line with previous reports [14–16]. The MSC surface phenotype characterized by expression of CD44, CD73, CD90, CD105, CD146, and CD166 was not significantly influenced by the treatments (Supplementary Fig. 2).

### Pre-treatment of MSCs with GDF-11 and RAP-536 affects subsequent HSPC distribution

Since functional alterations of MSCs have a significant impact on MDS progression [15], we investigated the potential for RAP-536 treatment of stromal cells to alter their subsequent support of normal hematopoiesis. To this end, we used MSC/HSPC in vitro co-cultures in which the stromal layers were pre-treated with combinations of GDF-11 and RAP-536 for 1 week before freshly isolated CD34+ cells from healthy donors were added (see Supplementary Fig. 1 for the experimental workflow).

**Fig. 2:**
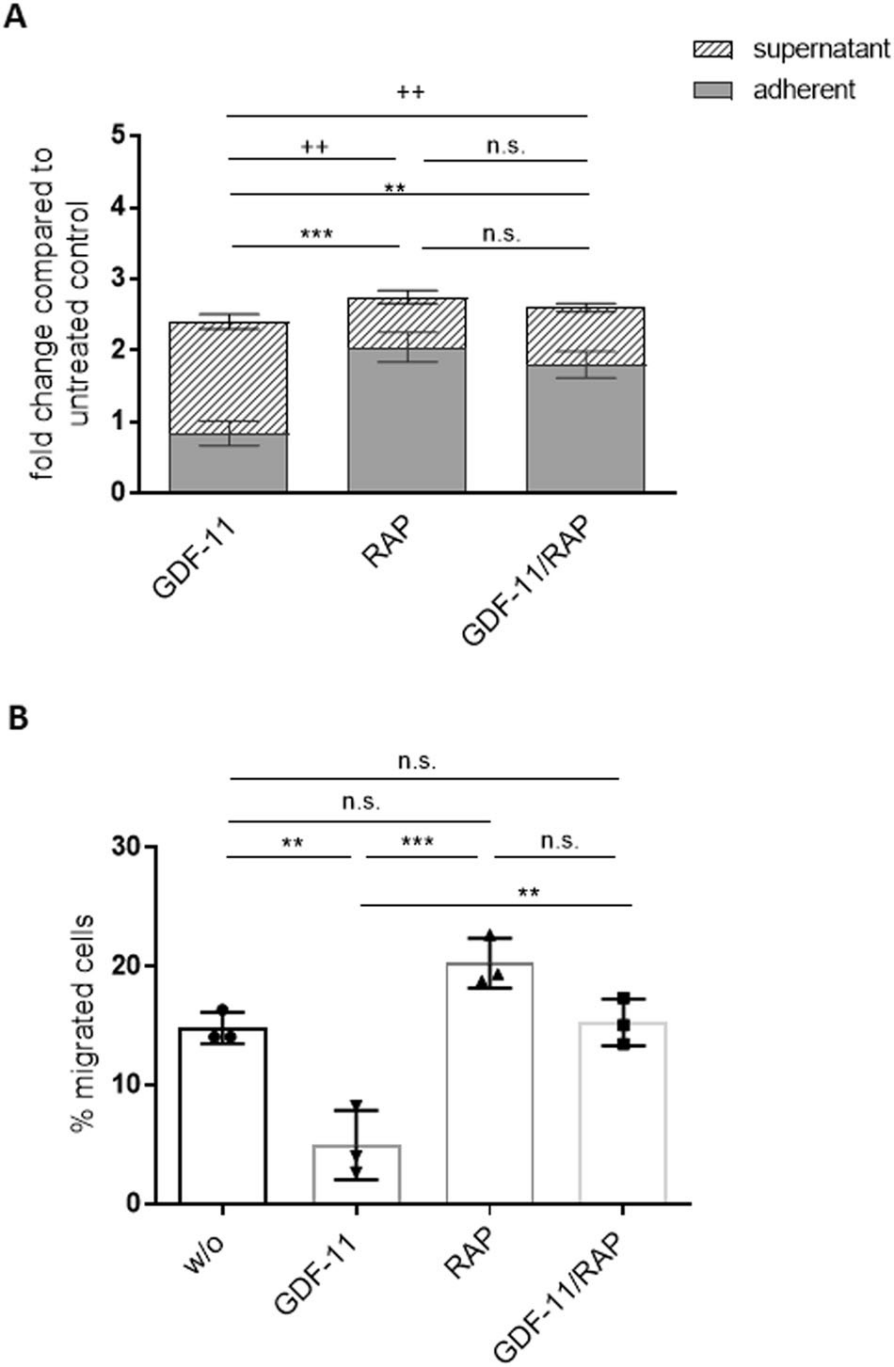
Pre-treatment of MSCs influences the HSPC migration and distribution in co-cultures. **A** After 1 week of co-culturing HSPCs derived from healthy donors on GDF-11/RAP-536 pre-treated MDS MSC layers, the fold change of CD45+ cell numbers compared to the untreated control layers was determined in the supernatant and the adherent fraction under the indicated treatment conditions. Error bars represent mean ± SD of the adherent fraction, ns *p* > 0.05, **p < 0.01, ***p < 0.001, and the supernatant fraction, ns *p* > 0.05, ^++^*p* < 0.01, *N* = 5, analyzed by two-way ANOVA with Tukey’s multiple comparisons test. **B** The migratory capacity of co-culture-derived HSPCs was analyzed in a Boyden chamber trans-well system. Error bars represent mean ± SD. ns *p* > 0.05, **p < 0.01, ***p < 0.001, *N* = 3 different co-culture experiments. Significance was assessed by one-way ANOVA with Tukey’s multiple comparisons test.

Strikingly, the number of non-adherent CD45+ cells after 7 days of co-culture was significantly increased by GDF-11 pre-treatment and decreased by RAP-536 pre-treatment of MDS MSC layers (Fig. 2A). In line with this, we detected significantly lower numbers of adherent CD45+ cells on GDF-11 pre-treated and higher numbers on RAP-536 pre-treated MSCs (Fig. 2A). This suggests that RAP-536 treatment of MSCs supports the subsequent interaction of HSPCs with the stromal layer.

To examine direct effects on HSPC migration, we performed trans-well migration Boyden chamber assays for CD45+ cells after 1 week of co-culture on pre-treated MDS MSC layers. HSPCs derived from the supernatant of RAP-536 primed stromal layers displayed significantly higher migration rates than those derived from untreated or GDF-11 pre-treated MSCs (Fig. 2B). Furthermore, freshly isolated HSPCs showed increased migration toward supernatants derived from RAP-536-treated MSCs and decreased migration toward those derived from GDF-11-treated MSCs (Supplementary Fig. 3).

### MSC-mediated effects on HSPC migration in vivo are reversed by RAP-536

**Fig. 3:**
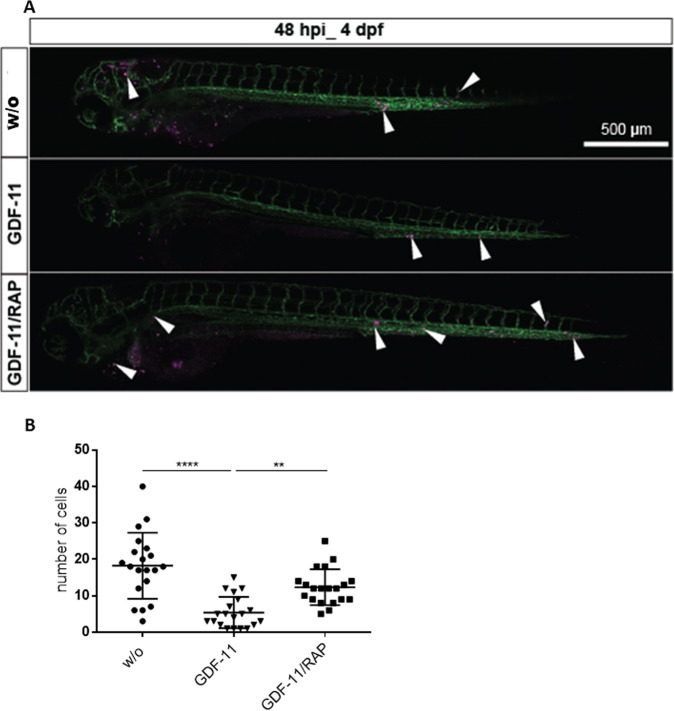
RAP-536 pre-treatment of MSCs enhanced HSPC migration potential in zebrafish. **A** Representative images of zebrafish larvae at 48 hpi. Addition of RAP-536 to GDF-11 pre-treatment of co-cultured MSCs rescued the survival and homing of HSPCs in the CHT region. Surviving HSPCs (magenta) are indicated by white arrowheads, the vasculature is labeled in green. Scale bar 500 µm. **B** Quantification of HSPCs in the tail region at 48 hpi. One representative experiment is shown, *N* = 20 embryos in each group, **p < 0.01, ****p < 0.0001. Significance was assessed with Kruskal–Wallis test followed by Dunn’s multiple comparisons post-hoc test.

In order to validate our findings in vivo, we used an embryonic zebrafish model in which cells injected into the duct of Cuvier can be monitored directly for in vivo migration and homing [17, 18]. Human cells persist in this model, because the adaptive immune system of the zebrafish is non-functional for the first 4 weeks post fertilization [13]. Hundred human HSPCs from 7 day co-cultures with GDF-11/RAP-536 pre-treated MDS MSCs were labeled with the vital fluorescent dye CM-Dil and injected intravenously in 48-h-old embryos. Live imaging of the transplanted cells via fluorescent confocal microscopy was performed 2 days post injection. Whereas HSPCs from GDF-11-treated MSCs were barely detectable in the zebrafish circulation, cells from RAP-536-treated stromal layers were present in high numbers in all regions of the embryo. These cells also showed increased migratory activity, especially into the tail region (Fig. 3A and Supplementary Videos 1 and 2). Quantification of HSPCs in embryonic xenografts revealed that cells primed on GDF-11 pre-treated MSCs persisted at significantly lower levels in vivo, confirming the inhibitory effect of these conditions. In contrast, cells that had been co-cultured on MSCs pre-treated with both GDF-11 and RAP-536 generated significantly higher numbers in the tail region, confirming the ability of RAP-536 to reverse the suppressive effects of GDF-11 (Fig. 3B).

### RAP-536 treatment of stromal cells restores hematopoietic expression of CXCR4 and CD61

Since the migration of HSPCs to and within the stromal microenvironment is directed mainly by the interaction of SDF-1 with its receptor CXCR4, we measured the expression of CXCR4 in the adherent and non-adherent HSPC fractions and found that the proportion of CXCR4-positive cells was consistently higher in the adherent fraction.

**Fig. 4:**
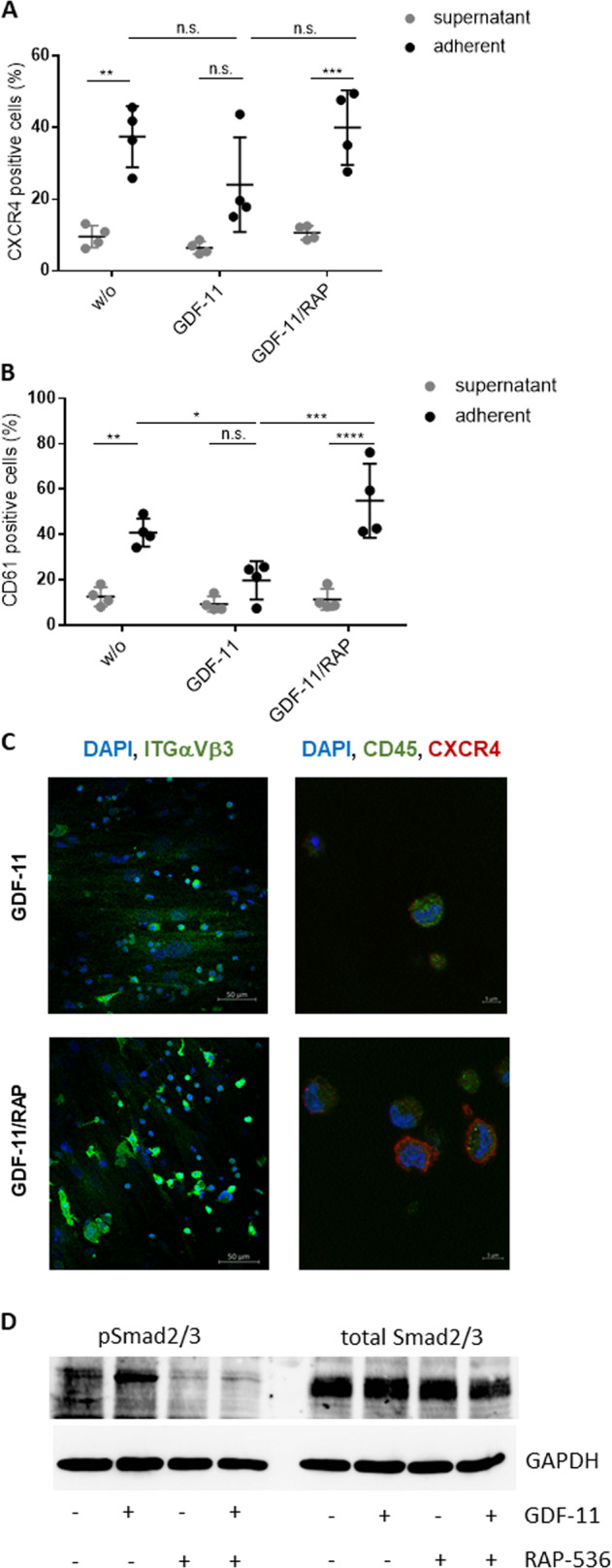
Phenotypical characterization of co-cultured HSPCs. HSPCs cultured for 1 week on GDF-11/RAP-536 pre-treated MDS MSC layers were evaluated by flow cytometry for the expression of **A** CXCR4 and **B** CD61. Cumulative data from four experiments are shown as mean ± SD. ns, *p* > 0.05, *p < 0.05, **p < 0.01, ***p < 0.001, ****p < 0.0001. Significance was assessed by two-way ANOVA with Sidak’s and Tukey’s multiple comparisons test. **C** Representative confocal microscopy images of ITGαVβ3 (green) and CXCR4 (red)/CD45 (green) with nuclear DAPI staining of HSPCs adherent on GDF-11 or GDF-11/RAP-536 pre-treated MDS MSC layers. **D** Western blot analysis of phospho Smad2/3 (52 kDa) and total Smad2/3 protein (52 kDa) in GDF-11/RAP-536 pre-treated MSCs after co-culture with HSPCs. Duplicate sample aliquots were run on a single gel, which was blotted before being cut into two halves: one being used for phosphor Smad2/3 and one for total Smad2/3 detection. GAPDH (37 kDa) served as reference protein. One representative experiment is shown, *N* = 3.

Although there was an apparent reduction in the proportion of CXCR4-positive adherent hematopoietic cells following GDF-11 pre-treatment of the MSCs, this did not reach significance (Fig. 4A). However, GDF-11 pre-treatment of MSCs did reduce subsequent CXCR4 positivity in the adherent cell fraction sufficiently to negate the clear and significant difference seen between the adherent and supernatant cells following co-culture on either untreated or GDF-11 + RAP-536-treated MSCs (Fig. 4A). This suggests that GDF-11 treatment weakens the SDF-1/CXCR4-dependent interaction between MSCs and HSPCs, while RAP-536 restores this activity.

While SDF-1/CXCR4 interactions are expected to attract HSPCs to the stromal layer, longer term retention is likely to be influenced by more direct cell–cell and cell–matrix interactions. Indeed, an analysis of integrin expression revealed a higher proportion of CD61 (integrin β3) positive cells in the adherent compared to the supernatant HSPC fractions in co-cultures with stromal layers (Fig. 4B). Furthermore, integrin ß3 expression on adherent HSPCs was clearly decreased by pre-treatment of the stromal layer with GDF-11 and restored by the inclusion of RAP-536. Neither treatment had a marked effect on the supernatant fraction (Fig. 4B) or on HSPCs in monoculture (not shown).

Immunofluorescence staining of HSPC co-cultures on GDF-11 ± RAP-536 primed MSC layers confirmed enhanced expression of both integrin αvβ3 and CXCR4 in HSPCs attached to RAP-536-treated MDS MSCs (Fig. 4C).

To confirm the sustained effect of GDF-11/RAP-536 pre-treatment of MSCs after co-culture with HSPCs, we once again analyzed Smad2/3 phosphorylation. Consistent with results shown above (Fig. 1A), GDF-11 pre-treatment of MSCs resulted in increased phosphorylation of Smad2/3 that was maintained after 1 week of HSPC co-culture. The addition of RAP-536 attenuated this effect. The total protein Smad2/3 level remained unaffected by either treatment (Fig. 4D).

### RAP-536/luspatercept treatment affects the SDF-1 level in vitro and in vivo

**Fig. 5:**
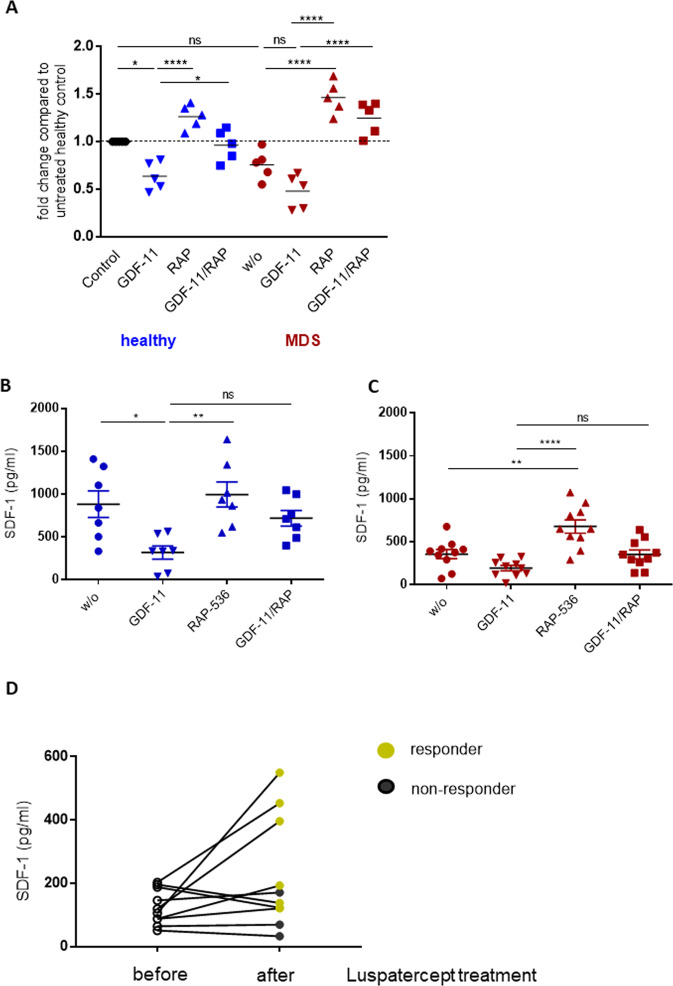
The SDF-1 levels in MSCs are modulated by GDF-11 and RAP-536 treatment. **A** mRNA quantification of healthy and MDS MSCs by real-time PCR. Relative target quantity was determined using the comparative CT (∆∆CT) method. Amplicons for SDF-1 were normalized to endogenous GAPDH expression and the untreated healthy MSCs were set to 1 (=control). Cumulative data from five donors are shown as mean ± SD. Significance was assessed by one-way ANOVA with Tukey’s multiple comparisons test. ns, *p* > 0.05, *p < 0.05, ****p < 0.0001. **B**, **C** SDF-1 protein levels in the MSC culture supernatant were analyzed by ELISA. Cumulative data from seven healthy donors and ten MDS patients are shown as mean ± SD. Significance was assessed by one-way ANOVA with Tukey’s multiple comparisons test. ns, *p* > 0.05, *p < 0.05, **p < 0.01, ****p < 0.0001. **D** SDF-1 levels were determined in bone marrow plasma of patients before and after treatment with luspatercept (*N* = 10). Black symbols indicate patients who did not respond to the treatment, green symbols the responders.

The fact that GDF-11 treatment of stromal cells reduces both migration/homing capacity of co-cultured HSPCs and CXCR4 expression in the stroma-associated fraction of HSPCs implies that a disruption of SDF-1 signaling may make an important contribution to the MDS phenotype. We found the expression of SDF-1/CXCL12 mRNA to be reduced by GDF-11 and restored by RAP-536 treatment in both healthy donor and MDS-derived MSCs (Fig. 5A). In line with previous reports, the SDF-1 baseline expression levels were lower in MDS MSCs compared to healthy donor MSCs [14, 15].

This was confirmed at the protein level by ELISA of MSC supernatants. Whereas healthy donor MSCs secreted a mean of 883.1 ± 411.5 pg/ml SDF-1 (Fig. 5B), MDS MSCs produced a far lower mean level of 356.5 ± 170.9 pg/ml (Fig. 5C). RAP-536 treatment of MSC cultures resulted in increased SDF-1 protein secretion, almost doubling the production from MDS MSCs (Fig. 5C). GDF-11 therefore decreased the production of SDF-1 at both the RNA and protein level, while the addition of RAP-536 reversed this effect.

There are likely to be TGF-ß family ligands other than GDF-11 that can target SDF-1 expression and be sequestrated by RAP-536. Indeed, the apparent overshoot effects of RAP-536 treatment on CD61 expression seen in Fig. 4 would be consistent with the involvement of further TGF-ß superfamily members in limiting hematopoietic support by MDS MSCs. To investigate this, we analyzed SDF-1 expression in MDS MSC cultures after treatment with recombinant GDF-8, GDF-15, and TGF-β in the presence or absence of RAP-536. Each of these factors led to a decreased SDF-1 secretion, which was ameliorated by the addition of RAP-536 (Supplementary Fig. 4A). Moreover, we confirmed these effects in co-cultures of pre-treated MDS MSCs with HSPCs, in which SDF-1 staining intensity and HSPC adhesion were both restored by priming the stromal layers with RAP-536 (Supplementary Fig. 4B).

We also determined the SDF-1 levels in bone marrow plasma before and after luspatercept treatment of MDS patients carrying SF3B1 mutation. Although there is a high variability in the detected SDF-1 concentration, we found an almost 50% increase in mean level of SDF-1 in the post-treatment samples (from 125.2 ± 46.6 to 224.8 ± 167.4 pg/ml), the highest levels being seen in responders [9] (Fig. 5D). In patients with a poor treatment response (“non-responders”) the SDF-1 plasma levels tended to remain low.

### RAP-536 rescues functional hematopoietic defects associated with co-culture on MDS stroma

**Fig. 6:**
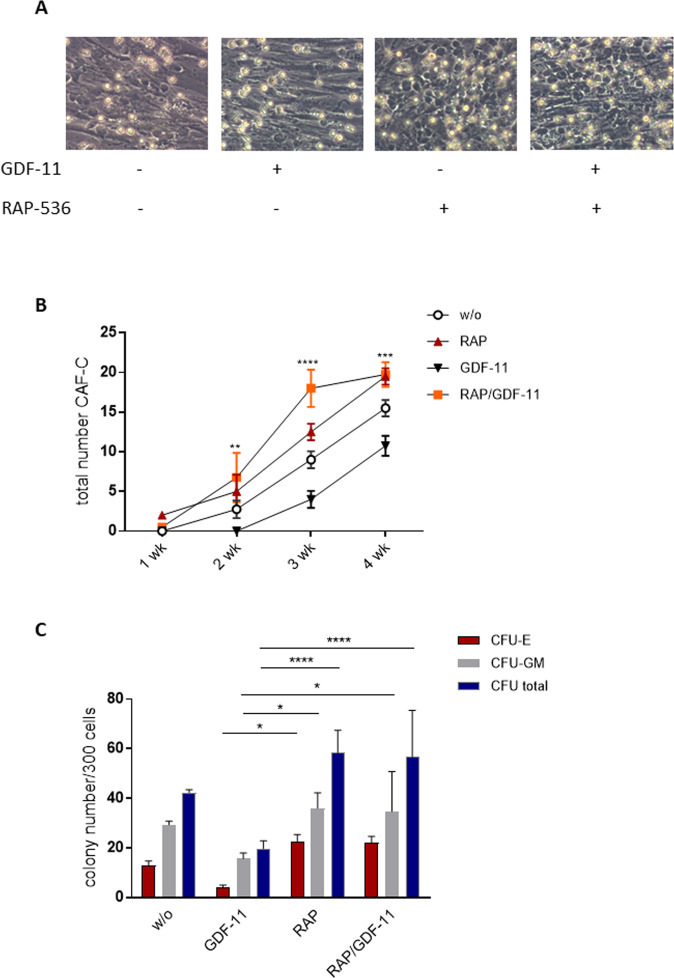
Hematopoietic support is inhibited by GDF-11-treatment of MSCs and restored by RAP-536. MDS MSCs were pre-treated with 0.1 µg/ml GDF-11 and/or 10 µg/ml RAP-536 for 7 days and co-cultured with freshly isolated CD34 + HSPC. **A** Representative images of CAF-C for each condition. **B** Quantification of CAF-C after 1, 2, 3, and 4 weeks. Pooled data from *N* = 3 MDS MSC/HSPC co-culture experiments are shown as mean ± SD, **p < 0.01, ***p < 0.001, ****p < 0.0001 for GDF-11 vs. RAP/GDF-11. **C** After 1 week of co-culture, a CFU assay was performed for 14 days in methylcellulose medium and the colonies were classified under the microscope or by using the StemVision system. Cumulative data from four experiments are shown as mean ± SD, *p < 0.05, ****p < 0.0001 by two-way ANOVA with Tukey’s multiple comparisons test. .

Given the sustained effects of RAP-536 on MSC phenotype in vitro and of luspatercept on the balance of hematopoietic activity in vivo, it was of interest to assess the effects of RAP-536 on the establishment of cobblestone areas, compact, stroma-associated colonies formed by long-term culture initiating cells. Cobblestone areas were evaluated in the co-culture system over 4 weeks and the results are shown in Fig. 6A, B. Pre-treatment of the MSCs with GDF-11 clearly reduced support capacity, with no cobblestone areas being apparent within the first 2 weeks and only a limited number appearing thereafter. In stark contrast, RAP-536 pre-treated MSCs provided improved functional support, increasing the number of cobblestone areas compared to untreated MSCs after just 1 week. Furthermore, RAP-536 rescued the inhibitory effect of GDF-11 seen at the 2-week time point. In general, the effects of pre-treatment were strongly diminished by the 4-week time point, suggesting that GDF-11 delays the establishment of cobblestone areas while RAP-536 overcomes this delay. Nonetheless, a significant advantage of RAP-536 pre-treated MSCs in terms of hematopoietic support was still apparent after this time (Fig. 6B).

To see whether the stromal effects of GDF-11 and RAP-536 extend to the level of proliferative differentiation and maturation, hematopoietic cells were removed from co-culture on un- or pre-treated healthy and MDS MSC monolayers after 1 week and introduced into hematopoietic colony forming assays. Consistent with the results shown above, GDF-11 pre-treatment of MDS MSCs reduced the subsequent colony forming capacity of co-cultured HSPCs compared to those cultured with untreated MDS MSCs (total colonies 19.7 vs. 42.3). This was largely due to a reduction in erythroid (4.0 vs. 13.0) rather than myeloid (15.7 vs. 29.3) colonies (Fig. 6C). The addition of RAP-536 to the GDF-11 pre-treatment of MSCs restored CFUs of all lineages (erythroid: 22.0; myeloid: 34.7, Fig. 6C), showing that the RAP-536 promotion of colony forming potential is not limited to the erythroid compartment. Of note, the number of CFUs was not significantly influenced by GDF-11/RAP-536 exposure of the HSPCs alone, demonstrating that the effects are predominantly mediated via the stromal layer (Supplementary Fig. 5).

### Luspatercept treatment in a clinical setting stably increases the stromal support for normal hematopoiesis

The MSC-HSPC co-culture system employed here identifies stromal cells as a primary target of both GDF-11 and RAP-536. To assess the relevance of stroma-mediated effects in the clinical situation, we determined the HSPC support capacity of MSCs isolated from MDS patients both before and after luspatercept treatment.

**Fig. 7:**
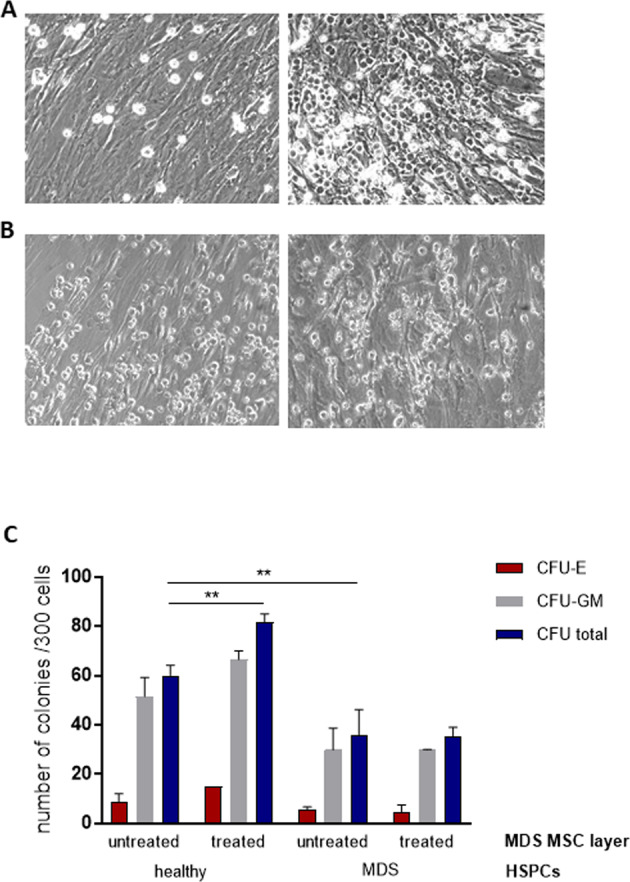
The stromal improvement by luspatercept in vivo supports normal but not MDS HSPCs. Representative images of co-cultures of MDS MSCs before (left) and after luspatercept treatment (right) with HSPCs from healthy donors (**A**) or the same MDS patient (**B**) at day 7. **C** After 1 week of co-culture, a CFU assay was performed for 14 days in methylcellulose medium and the colonies were classified using the StemVision system. Data are shown from one experiment in duplicate as mean ± SD, **p < 0.01 by two-way ANOVA with Tukey’s multiple comparisons test.

Normal HSPCs showed higher levels of adhesion and proliferation than MDS HSPCs especially on post-treatment MSCs (Fig. 7A, B). Moreover, MDS MSCs derived after luspatercept treatment supported an increased cobblestone area formation of healthy but not MDS HSPCs (Fig. 7A, B).

**Fig. 8:**
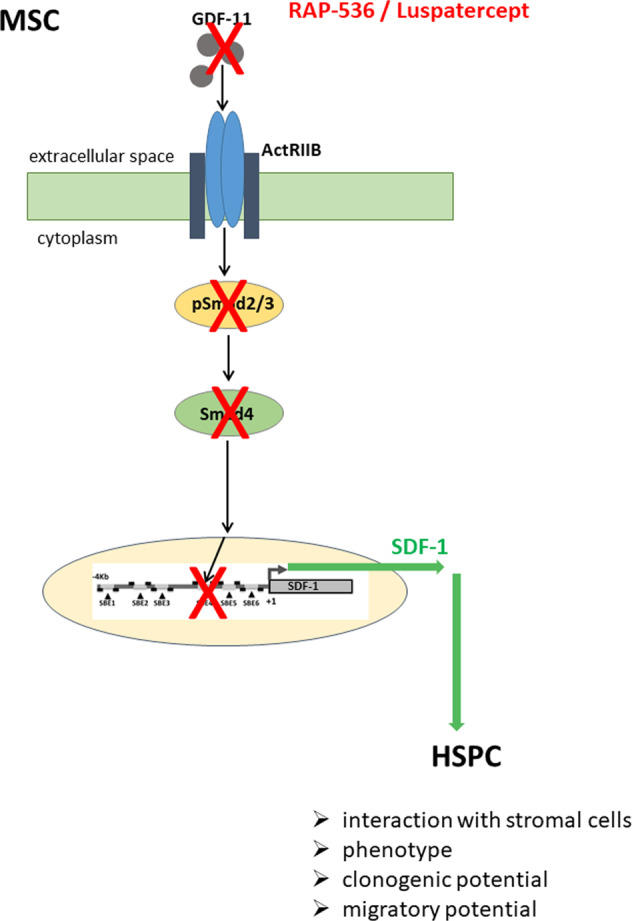
Graphical summary. High GDF-11 levels in the MDS BMME activate Smad2/3 phosphorylation and expression of Co-Smad4 that binds to the SDF-1 promoter region [7] thereby suppressing transcription and leading to impaired hematopoiesis. Trapping GDF-11 by RAP-536/luspatercept reduces Smad2/3 and Smad4 activity resulting in SDF-1 promoter activation and elevated expression, thereby restoring HSPC support.

Importantly, colony forming assays performed with cells removed after 1 week of co-culture showed that in in vivo luspatercept treatment resulted in a stable increase in the ability of bone marrow-derived stroma to support normal hematopoiesis, without affecting the degree of support for MDS-derived HSPCs (Fig. 7C). This suggests a robust, stromal-mediated effect of luspatercept that rescues the defective support for normal hematopoiesis that is characteristic of MDS stroma (Fig. 8).

## Discussion

Luspatercept is a novel fusion protein that blocks TGF-β superfamily ligands, such as GDF-11 and activin B, thereby restoring late-stage erythropoiesis in anemic lower-risk MDS patients [8, 9]. Although the involvement of the BMME in MDS disease development and progression is clearly documented [3, 16, 19, 20], the potential influence of luspatercept on the stromal niche compartment is unknown. Here, we used an in vitro co-culture system to provide clear evidence for an impact of the luspatercept analog RAP-536 on the stromal microenvironment in MDS. Treatment of both MDS and healthy MSCs with RAP-536 reversed the GDF-11-dependent Smad2/3 activation and the accompanying increase in Smad4 levels. The restoration of hematopoietic support by RAP-536 in vitro and luspatercept in vivo is conducted by an increase in SDF-1 expression.

The regulation of normal hematopoiesis is facilitated by stroma-derived cytokines, the correct balance of stimulating and suppressive signals being required for optimal production of the various hematopoietic cell lineages [21]. Circulating HSPCs are attracted to stromal cells mainly via CXC-receptors interacting with SDF-1. In turn, activation of these receptors is known to induce the expression of integrins that promote endothelial adhesion and trans-endothelial migration [22]. We found CXCR4, the most common SDF-1 receptor, to be more widely expressed in the HSPC population associated with RAP-536 primed stromal layers. Our demonstration of an increased proportion of CD61 positive cells in the adherent population following RAP-536 treatment of the stroma suggests that SDF-1-mediated induction of integrin αVβ3 may result in more robust retention of HSPCs in the niche [23].

CD61 (β3 integrin) has been shown to be a positive marker for enrichment of LT-HSCs and may contribute to the general determination of stemness defined by niche interactions, self-renewal, and quiescence [24]. Indeed, we detected significantly higher CAF-C and CFU activity in HSPCs co-cultured on RAP-536-treated MSC layers, suggesting that CD61+ cells may give rise to the colonies. The clonogenic capacity of HSPCs was not affected differentially by the source of the MSCs (MDS or healthy) used in the co-culture. Previous studies using LTC-IC/CFU assays to investigate the hematopoietic support by MDS-derived MSCs have yielded conflicting results in this respect, most likely due to experimental differences [25–27]. Of note, our CFU results show that the blockage of GDF-11 by RAP-536 in MSC/HSPC co-cultures can restore support for both erythroid and myeloid progenitors. This is in line with the observation in a MDS PDX-murine model that RAP-536 treatment resulted in higher levels of white blood cells as well as increased hemoglobin [28]. Moreover, patients enclosed in the MEDALIST trial (NCT02631070), a phase 3 study evaluating the efficacy and safety of luspatercept, demonstrated increased levels of both neutrophils and platelets after receiving luspatercept [29]. Our experiments using co-cultures with MSCs from patients who had received luspatercept showed improved support for the co-cultured normal but not MDS HSPCs in terms of CAF-C and CFU numbers. This is in line with the clinical observation that the allelic burden of SF3B1 and other MDS-associated genes is rather not changed during luspatercept treatment suggesting the absence of a disease-modifying activity.

Tracking of previously co-cultured HSPCs in embryonic zebrafish confirmed our in vitro data showing that GDF-11 suppresses the hematopoietic support of MSCs and that this can be rescued by RAP-536. Specifically, we found a significantly higher number of HSPCs in the embryonic zebrafish when these cells had been primed on a GDF-11/RAP-536-treated MSC layer. Interestingly, it has been demonstrated in a trans-well assay that human HSPCs appear to respond to zebrafish-derived chemotactic cues such as SDF-1 [13]. It is therefore feasible that improved function in the zebrafish environment is due at least in part to a more effective response to zebrafish factors such as SDF-1 following transplantation.

We found no evidence of a direct effect of RAP-536 on HSPCs, either at the level of integrin or CXCR4 expression or in terms of increase hematopoietic activity, emphasizing the important role of the stromal microenvironment. The conclusion that impaired stromal support contributes to the ineffective hematopoiesis and peripheral cytopenias observed in patients with MDS is further supported by the effects of ACE-011 (sotatercept), an activin receptor type IIA antagonist, which has been shown to be mediated via stromal cells and not directly by HSPCs [30].

An association of the functional impairment of the BMME in MDS and reduction in SDF-1 expression has previously been noted by us and others [14, 15, 31]. This suggests that targeted upregulation of the SDF-1/CXCR4 axis might support the remaining normal hematopoiesis, increasing the ability of normal hematopoietic progenitors to compete with the malignant clone that is likely to be a particularly important issue during the early stages of MDS. We therefore chose to analyze the effects of RAP-536 on SDF-1 in more detail. In line with the changes in gene expression detected after GDF-11/RAP-536 in vitro treatment of MSCs, we confirmed corresponding changes in SDF-1 protein secretion. We have previously described the restoration of SDF-1 levels in MSCs achieved by blocking TGF-β with inhibitory antibodies [32]. This suggests a direct or indirect modulation of SDF-1 via the Smad signaling pathway. Indeed, six Smad binding elements have been identified upstream of the transcription start site of the SDF-1 gene [7]. Deletion of the Smad binding elements responsible for Smad4 binding led to an increase in basal expression levels of SDF-1, confirming suppression of SDF-1 transcription by Smad signaling [7]^.^ While this provides a rationale for the cell-intrinsic modulation of SDF-1 transcription in response to TGFß ligands, it remains to be seen whether the GDF-11-mediated functional inhibition of MSCs in MDS in vivo is a cell-intrinsic mechanism or is extrinsically mediated by other cells within the BMME [33].

Our observation that RAP-536 targets SDF-1 expression in stromal cells and improves hematopoiesis identifies a potential mechanism but has limitations due to the in vitro setting. To assess the clinical relevance, we determined the SDF-1 levels in bone marrow plasma from a small cohort of MDS patients with SF3B1 mutation before and after receiving luspatercept [10] and detected a tendency for increased levels of SDF-1 post treatment that was most notable in the clinical responders. Analysis of a larger patient cohort and validation of our results will be necessary to assess the potential of SDF-1 as a biomarker in this context.

It is important to note that RAP-536/luspatercept may trap not just GDF-11, but a range of related ligands such as GDF-8, GDF-15, and TGF-β, each of which may be involved in the altered hematopoiesis in MDS. In our in vitro setting, we demonstrate that RAP-536 is able to mitigate the effects of these molecules to various extents by increasing the SDF-1 secretion, which in turn increased the number of HSPCs adhering to the pre-treated MSC layers. Indeed, recent publications have even excluded GDF-11 as the main target of RAP-536 based on genetic deletion of GDF-11 in a murine model [34, 35]. Further work will be required to determine the true degree of redundancy and compensation between related factors in the disease setting. However, it is already evident that GDF-11 is just one of multiple Smad2/3 pathway ligands collectively regulating erythropoiesis in mice [36].

In summary, our data provide the first evidence that RAP-536/luspatercept targets MSCs from patients with MDS. Trapping of GDF-11 prevents the over-activation of Smad2/3 and is likely to improve hematopoiesis by restoring suppressed expression of SDF-1 in MDS.

## Supplementary information

Supplementary Data

Supplementary Figure 1

Supplementary Figure 2

Supplementary Figure 3

Supplementary Figure 4

Supplementary Figure 5

Dissemination of GDF-11-treated HSPCs in vivo.

Improved migration potential of GDF-11/RAP-536-treated HSPCs.
