# Supplementary material for: Luspatercept restores SDF-1-mediated hematopoietic support by MDS-derived mesenchymal stromal cells
Source: Leukemia. 2021 May 17;35(10):2936–47. doi: 10.1038/s41375-021-01275-5 (PMC8478655; doi:10.1038/s41375-021-01275-5)
Supplement: Supplementary file 1 — Supplementary Data [file 41375_2021_1275_MOESM1_ESM.docx]

**Luspatercept restores SDF-1-mediated hematopoietic support by MDS-derived mesenchymal stromal cells**

Manja Wobus, Anna Mies, Nandini Asokan, Uta Oelschlägel, Kristin Möbus, Susann Winter, Michael Cross, Heike Weidner, Martina Rauner, Lorenz C. Hofbauer, Martin Bornhäuser, Uwe Platzbecker

**Supplementary information**

**Content:**

Supplementary methods incl. references

Supplementary figures

Figure S1. Schematic depiction of the experimental work flow for *in vitro* MSC/HSPC cocultures.

Figure S2. The MSC phenotype is not affected by GDF-11 and RAP-536 treatment.

Figure S3. Pre-treatment of MSCs influences the HSPC migratory activity.

Figure S4. The SDF-1 levels in MDS MSCs are inhibited by GDF-8, GDF-15 or TGFβ, respectively, and can be restored by RAP-536 treatment.

Figure S5. The clonogenic potential of HSPCs is not directly affected by GDF-11/RAP-536.

Supplementary Videos

Video S1. Dissemination of GDF-11-treated HSPCs *in vivo*.

Video S2. Improved migration potential of GDF-11/RAP536-treated HSPCs.

Supplementary Tables

Table S1. Patient’s characteristics.

Table S2. Antibodies used for flow cytometry.

Table S3. Primer sequences.

**Supplementary methods**

**Flow cytometry**

For flow cytometric analyses of cell surface markers on MSCs after treatment with 0.1 µg/ml GDF-11 and 10 µg/ml RAP-536, cells were trypsinized, suspended in PBS containing 5% BSA and stained with fluorescence-labelled antibodies as mentioned in Supplementary Table 2.

HSPCs from cocultures were stained for surface markers using fluorescently labeled antibodies according to Supplemental Table 2. Corresponding human immunoglobulin G controls were used. Data was acquired on a BD FACS Calibur or LSRII. Data were analysed using FlowJo software (FlowJo, LLC).

**Western blot**

Whole cell lysates were prepared using RIPA lysis buffer containing proteinase inhibitors. Protein concentrations were determined using the BCA Protein Assay Kit (Thermo). Equal protein amounts were separated by SDS-polyacrylamide gel electrophoresis and transferred to polyvinylidene ﬂuoride membranes (Bio-Rad Laboratories) for detection of speciﬁc proteins using primary antibodies for Phospho-Smad2/Smad3 (Cell Signaling), Smad4 (Thermo) and GAPDH (Abcam) overnight at 4°C, following secondary antibodies HRP-conjugated goat anti-mouse IgG (Invitrogen) or donkey anti-rabbit IgG (GE Healthcare), respectively. The blots were incubated with ECL Plus Western Blotting reagent (Amersham) and the signals captured using a LAS3000 imaging system.

**HSPC trans-well migration assay**

Trans-well migration toward the conditioned medium of either untreated or GDF-11/RAP-536-treated MSCs were performed as described previously ^1^. Briefly, 2 × 10^5^ HSPCs were added to the upper chamber in 0.1 ml of medium and allowed to migrate for 4 h at 37°C toward 600 µl of MSC-conditioned or fresh medium through a 5-µm polycarbonate membrane. Cells in the lower chamber were then counted using MACSquant.

**Animal handling and cell preparation for transplantation**

*Casper12* and *[Tg(kdrl:EGFP)^s843^* strains of zebrafish (*Danio rerio*) were kept under standard conditions (28ºC in E3 buffer) until 48 hpf. All animal experiments were conducted at larval stages before the point of independent feeding and were in keeping with the animal protection laws (Tierschutzgesetz). HSCPs were labeled using fluorescent cell tracker CM-DiI (Invitrogen) as described previously^3^, loaded in a glass capillary and micro-injected into the blood circulation via the duct of Cuvier (DoC)^4^*.* Engrafted embryos were transferred to a fresh petri dish and maintained at 33˚C. Based on the fluorescence spread of the injected embryos at 2 hours post injection (hpi), embryos with HSPCs in the blood circulation were selected for further experiments.

**Image acquisition and processing**

The migration of injected HSPCs was analyzed by live imaging of the engrafted embryos using a Dragonfly spinning disk microscopy system. The caudal hematopoietic tissue (CHT) region of injected embryos were imaged at 10x magnification at 10 minute intervals for a total of 12 h. Images were subsequently stitched and processed using Arivis Vision 4D and Image J plugins. For quantification, embryos were fixed in 4% paraformaldehyde at 4˚C overnight. Fixed embryos were imaged by inverted confocal microscopy (Zeiss LSM 780) at 20x magnification (whole embryos) or at 40x magnification (tail region). Confocal stacks were converted to maximum intensity projections using Image J (v 1.51h) and quantification of HSPC survival at 2 dpi performed manually.

**Real-time polymerase chain reaction (RT-PCR)**

RNA was isolated from MSC using RNeasy Mini Kit (Qiagen) and reverse transcribed into cDNA using RevertAid cDNA synthesis kit (Thermo) with oligo-dT primers. Relative target quantity was determined using the comparative CT (∆∆CT) method. RT-PCR was performed using SYBR Green/ROX PCR master mix (Thermo) and target specific primers (Supplemental Table 3) on a Taqman Fast 3500 cycler (Applied Biosystems). Amplicons were normalized to endogenous GAPDH control.

**Chemokine measurements in cell culture supernatants and bone marrow plasma**

Standardized 1-ml aliquots from culture supernatants were collected from healthy and MDS MSCs either untreated or treated with GDF-11 and/or RAP-536. Plasma samples were diluted 1:2 prior to the analysis. The protein levels of SDF-1 were quantified using an enzyme-linked immunosorbent assay (ELISA, R&D Systems) according to the manufacturer’s protocol.

**Immunofluorescence staining and confocal laser scanning microscopy**

Cocultures of pre-treated MSCs with adherent HSPCs were fixed with 4% paraformaldehyde. Cells were permeabilized using PBS containing 0.1% Triton X-100 (T-PBS), blocked with T-PBS containing 10% FCS and 1% HSA (IF-Buffer) and incubated over night at 4°C with antibodies as follows: monoclonal mouse-anti-human-ITGαVβ3 (Abcam) 1:200, polyclonal rabbit-anti-human-CXCR4 (Abcam) 1:500, monoclonal mouse-anti-human-CD45 (Miltenyi Biotec) 1:100. Secondary antibodies were incubated for 1 h at room temperature as follows: polyclonal sheep-anti-rabbit-Cy3 (Sigma-Aldrich) 1:200 or polyclonal goat-anti-mouse-Cy2 (Dianova). Cell nuclei were counterstained with DAPI (Sigma-Aldrich). Imaging analysis was performed by confocal microscopy (LSM800, Carl Zeiss) and ZEN software.

**Supplementary references**

1 Jing D, Wobus M, Poitz DM, Bornhäuser M, Ehninger G, Ordemann R. Oxygen tension plays a critical role in the hematopoietic microenvironment in vitro. *Haematologica* 2012; **97**: 331–339.

2 White RM, Sessa A, Burke C, Bowman T, LeBlanc J, Ceol C *et al.* Transparent Adult Zebrafish as a Tool for In Vivo Transplantation Analysis. *Cell Stem Cell* 2008; **2**: 183–189.

3 Asokan N, Daetwyler S, Bernas SN, Schmied C, Vogler S, Lambert K *et al.* Long-term in vivo imaging reveals tumor-specific dissemination and captures host tumor interaction in zebrafish xenografts. *Sci Rep* 2020; **10**. doi:10.1038/s41598-020-69956-2.

4 Jin S-W. Cellular and molecular analyses of vascular tube and lumen formation in zebrafish. *Development* 2005; **132**: 5199–5209.

**Supplementary figure legends**

**Figure S1.** Schematic depiction of the experimental work flow for *in vitro* MSC/HSPC cocultures.

**Figure S2. The MSC phenotype is not affected by GDF-11 and RAP-536 treatment.** Representative flow cytometry histograms for a MDS MSC sample displaying the expression of CD44, CD73, CD90, CD105, CD146 and CD166.

**Figure S3.** **Pre-treatment of MSCs influences the HSPC migratory activity.** The migratory capacity of freshly isolated HSPCs towards pre-treated MDS MSC layers was analyzed in a Boyden chamber trans-well system. Error bars represent mean ± SD. ns, p> 0.05, N=3 different coculture experiments.

**Figure S4. The SDF-1 levels in MDS MSCs are inhibited by GDF-8, GDF-15 or TGFβ, respectively, and can be restored by RAP-536 treatment. a** SDF-1 protein levels in the MSC culture supernatant were analyzed by ELISA. Cumulative data from 3 MDS patients are shown as mean ± SD. **b** Representative confocal microscopy images of SDF-1 (green) and CD45 (red) with nuclear DAPI staining of HSPCs adherent on GDF-8, GDF-15, TGFβ +/- RAP-536 pre-treated MDS MSC layers.

**Figure S5.** **The clonogenic potential of HSPCs is not directly affected by GDF-11/RAP-536.** CFU assays were performed using freshly isolated CD34+ HSPCs without coculture in the presence or absence of GDF-11/RAP-536 for 14 days in methylcellulose medium and the colonies were classified by using the StemVision system. N=4.

**Supplementary Videos**

**Video S1. Dissemination of GDF-11-treated HSPCs *in vivo***. Isolated HSPCs labeled with CM-DiI (magenta) were injected into embryonic zebrafish expressing the vasculature (green) marker *Tg(kdrl:EGFP)^s843^*. Time-lapse images were recorded 1 hour after injection focusing on the caudal hematopoietic tissue (CHT) of the tail region of the embryo. GDF-11-treated HSPCs migrated along the blood flow, however very few cells disseminated towards the CHT region, while others stayed close to the injected site. Time shown as h:min:sec. Scale bar: 100 µm.

**Video S2. Improved migration potential of GDF-11/RAP536-treated HSPCs**. GDF-11/RAP536-treated HSPCs labeled with CM-DiI (magenta) were injected into embryonic zebrafish expressing the vasculature (green) marker *Tg(kdrl:EGFP)^s843^*. After injection, HSPCs disseminated from head to tail. Time-lapse images on the caudal hematopoietic tissue (CHT) region of the embryo showed an increase in number of HSPCs migrating towards the tail. A few cells migrated as clusters seen adhering to the CHT site, one or two cells were enclosed inside endothelial cell in a “cuddling” context. Time shown as h:min:sec. Scalebar: 100µm

**Supplementary Tables**

**Table S1. Patient’s characteristics.**

| **No.** | **Sex** | **Age (years)** | **Diagnosis_WHO2016** | **Karyotype** | **Molecular genetics (Mut VAF %)** | **IPSS** | **IPSS-R** |
| --- | --- | --- | --- | --- | --- | --- | --- |
| 1 | m | 70 | CMML-1 | 46,XY | JAK2 (V617F 1%) | Int-1 | Int |
| 2 | f | 42 | MDS del5q | 46,XX, del5q |  | Int-1 | Int |
| 3 | f | 70 | MDS del5q | 46,XX, del(5)(q13q33)[25] |  | Low | Low |
| 4 | f | 72 | MDS/MPN-RS-T | 46,XX,t(1;3)(p36;q21)[16]/46,XX[4] | SF3B1 (K700E 25%) | Int-1 | High |
| 5 | m | 69 | MDS-RS-SLD | 46,XY[20] |  | Low | Low |
| 6 | f | 50 | MDS-EB-1 | 46,XX[26] |  | Int-1 | Int |
| 7 | f | 27 | MDS-RS-MLD | 46,XX |  | Low | Low |
| 8 | m | 59 | MDS-EB-1 | 46,XY,der(16)t(1;16)(q21;q12),del(20)(q11.2)[cp3]/46,XY[5] |  | Int-1 | Int |
| 9 | f | 73 | MDS-EB-2 | 46,XX,del(5)(q13q33)[16]/46,XX[6] |  | Int-1 | Int |
| 10 | m | 78 | MDS-EB-1 | 46,XY[25] |  | Int-1 | Int |
| 11 | m | 63 | MDS-MLD | 46,XY[11] | ASXL1 (E635R 19%) | Low | Low |
| 12 | m | 76 | MDS-RS-MLD | 46,XY[25] | DNMT3A (R882P 32%), SF3B1 (E622D 37%), TET2 (I1873T 21%) | Low | Low |
| 13 | m | 73 | MDS-EB-2 | 46,XY[26].nuc ish 3q26(MECOMx2)[200] | ASXL1 (G646W 20%), BCORL1 (R72W 99%), IDH2 (R140Q 45%), SRSF2 (P95H 34%) | Int-2 | High |
| 14 | f | 79 | MDS-RS-MLD | 46,XX |  | Low | Int |
| 15 | m | 67 | MDS-RS-MLD | 46,XY |  | Int-1 | Int |
| 16 | m | 59 | MDS-RS-MLD | 46,XY | SF3B1 (H662Y 20%) | Low | Low |
| 17 | m | 78 | MDS-EB-1 | 46,XY[26].nuc ish 3q26(MECOMx2)[200] |  | Int-1 | Int |
| 18 | m | 74 | MDS-RS-MLD | 47,XY,+19 |  | Int-1 | Int |
| 19 | m | 78 | MDS-EB-1 | 46,XY[25] |  | Int-1 | Int |

**Table S2. Antibodies used for flow cytometry.**

| **Target** | **host/isotype/conjugate** | **Company** |
| --- | --- | --- |
| CD34 | mouse IgG2aκ/APC | Miltenyi Biotec |
| CD45 | mouse IgG1/PE-Cy7 | BD Biosciences |
| CD61 | recombinant human IgG1/PE | Miltenyi Biotec |
| CD44 | mouse IgG1/APC | Miltenyi Biotec |
| CD73 | mouse IgG1k/PE | Miltenyi Biotec |
| CD90 | mouse IgG1/FITC | Miltenyi Biotec |
| CD105 | mouse IgG1k/APC | Miltenyi Biotec |
| CD146 | recombinant human IgG1/APC | Miltenyi Biotec |
| CD166 | mouse IgG1k/PE | BD Biosciences |
| CD184 | mouse IgG2α/PE | BD Biosciences |

**Table S3. Primer sequences.**

| **Gene** |  | **Sequence** |
| --- | --- | --- |
| GAPDH | forward  reverse | 5‘-GAAGGTGAAGGTCGGAGTC-3‘  5‘-GAAGATGGTGATGGGATTTC-3‘ |
| SDF-1 | forward  reverse | 5‘-GGTCCGTCCTGTCTTGATGT-3‘  5‘-ACTGGGTGTACCACCTGCTC-3‘ |
