# Supplementary figures and images for: Luspatercept restores SDF-1-mediated hematopoietic support by MDS-derived mesenchymal stromal cells
Source: Leukemia. 2021 May 17;35(10):2936–47. doi: 10.1038/s41375-021-01275-5 (PMC8478655; doi:10.1038/s41375-021-01275-5)

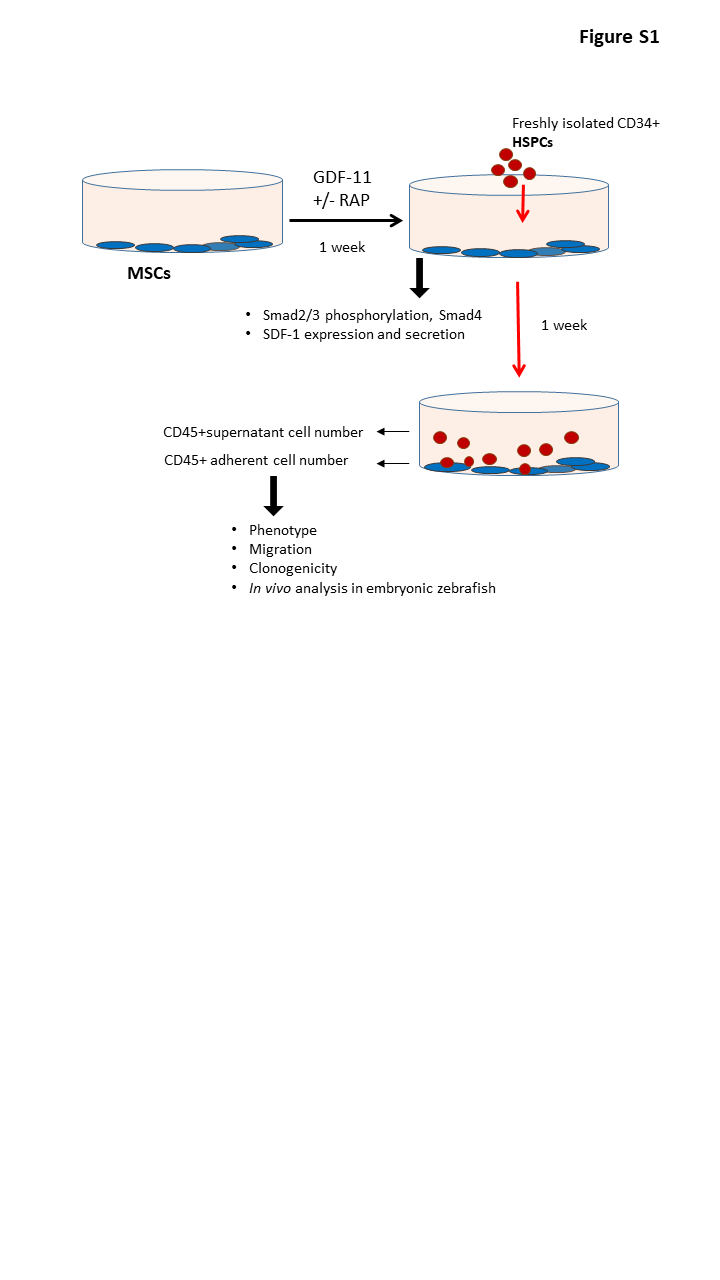

Supplement: Supplementary file 2 — Supplementary Figure 1 [file 41375_2021_1275_MOESM2_ESM.tif]

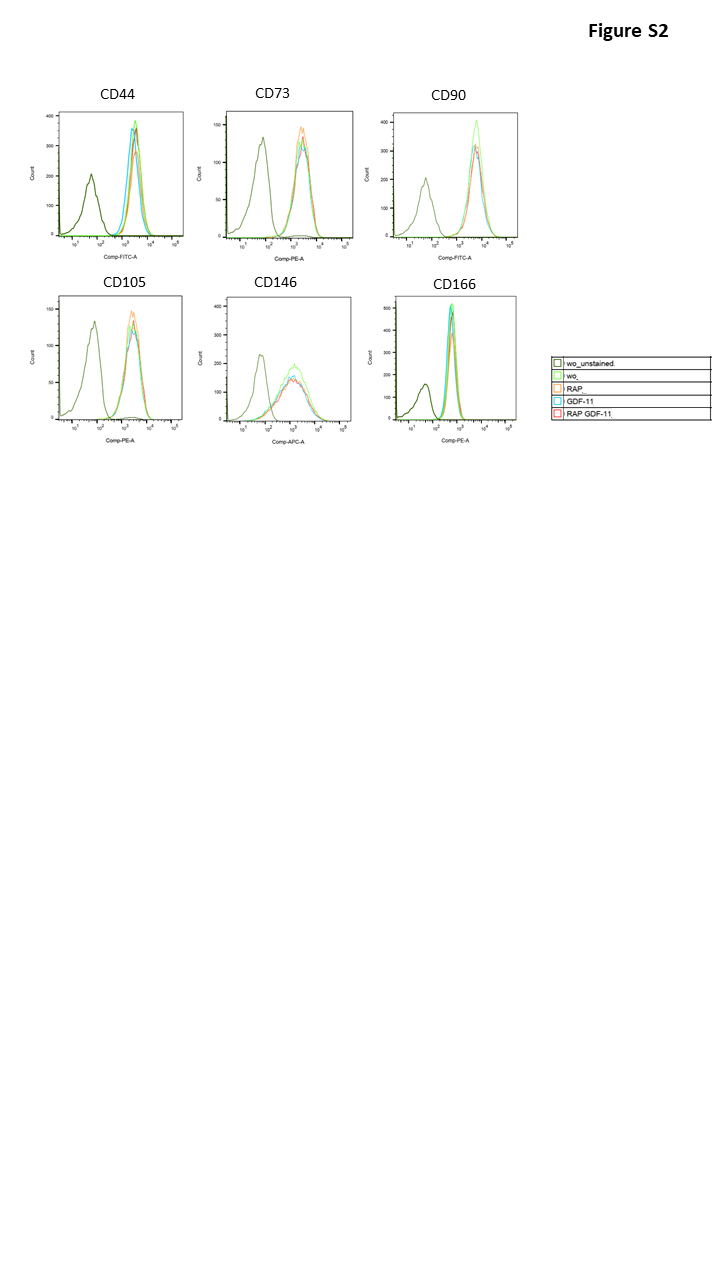

Supplement: Supplementary file 3 — Supplementary Figure 2 [file 41375_2021_1275_MOESM3_ESM.tif]

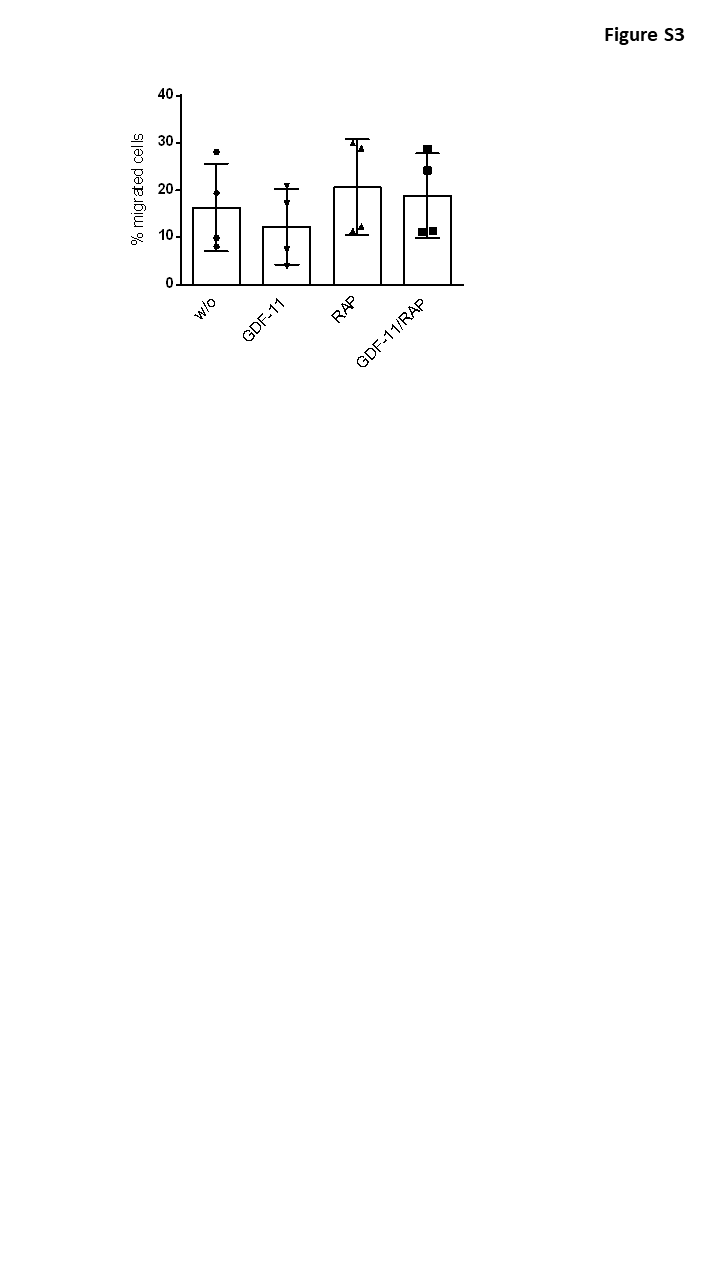

Supplement: Supplementary file 4 — Supplementary Figure 3 [file 41375_2021_1275_MOESM4_ESM.tif]

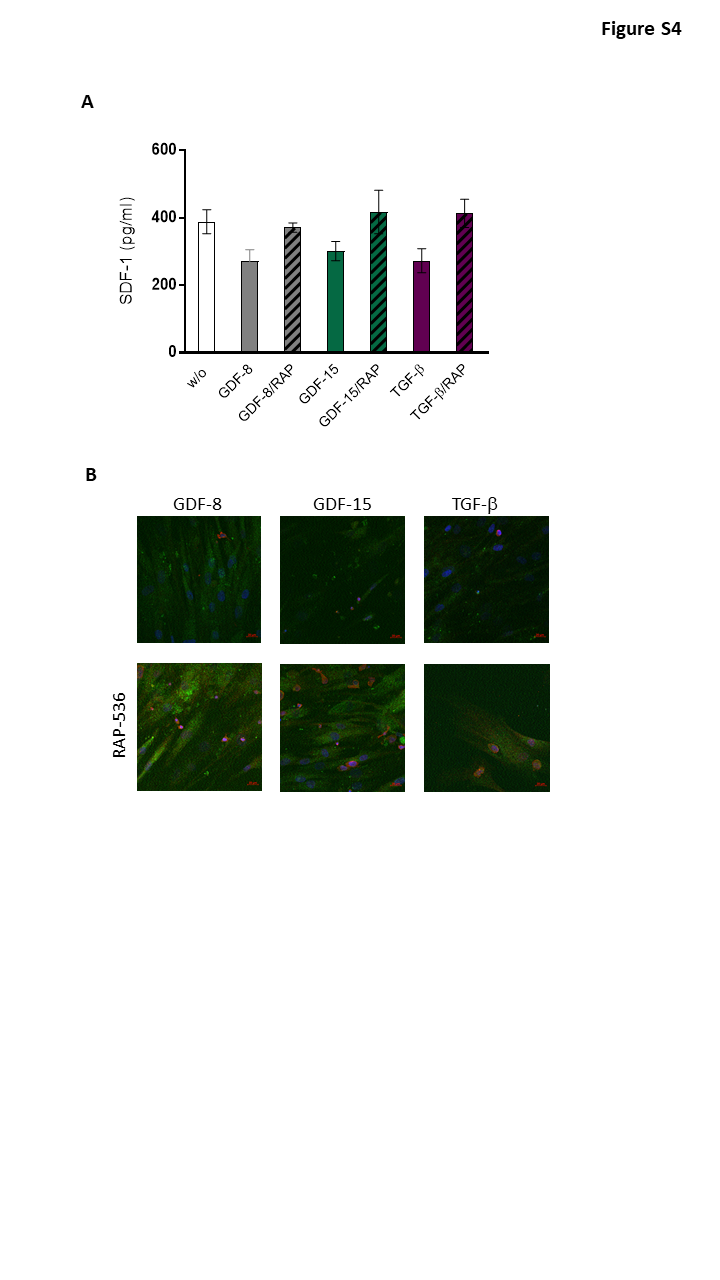

Supplement: Supplementary file 5 — Supplementary Figure 4 [file 41375_2021_1275_MOESM5_ESM.tif]

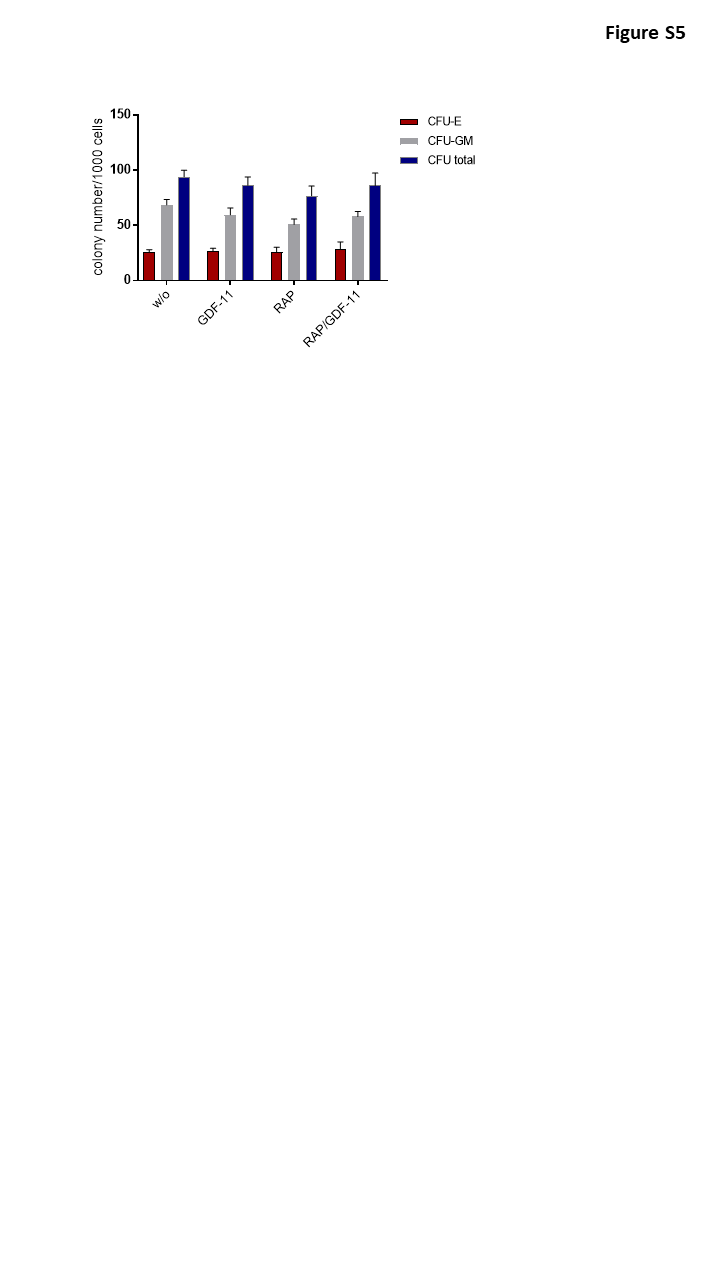

Supplement: Supplementary file 6 — Supplementary Figure 5 [file 41375_2021_1275_MOESM6_ESM.tif]
